# Surgical Technique – The “Derriere View” for Evaluation of Guidewire Position in Cephalomedullary Nailing of Intertrochanteric Femur Fractures

**DOI:** 10.7759/cureus.116189

**Published:** 2026-09-13

**Authors:** Omar El Dishish, Qing Hang Tan, Amirzeb Aurangzeb, Kevin Ho Yin Wong, Andy Yeo

**Affiliations:** 1 Orthopaedic Surgery, Changi General Hospital, Singapore, SGP

**Keywords:** cephalomedullary nailing, “derriere view”, evaluation of guidewire position, intertrochanteric femur fractures, surgical technique

## Abstract

Intertrochanteric (IT) hip fractures are common in the elderly and are frequently treated with cephalomedullary nailing. Despite advances in implant design, fixation failure, such as cut-out or cut-through, remains a concern, often related to suboptimal implant positioning and limited intraoperative visualization of the femoral neck cortex. This technical report describes the Derriere view, an additional intraoperative fluoroscopic projection applied during cephalomedullary nailing of intertrochanteric fractures with the targeting jig and instrumentation in situ.

The view is obtained by rotating the C-arm approximately 40° cranially from the standard lateral position and is intended to provide additional visualization of the posterior femoral neck cortex when this is inadequately delineated on conventional projections. The technique has been routinely used at our center as an adjunct to standard fluoroscopic views, particularly when conventional anteroposterior and lateral projections provide inadequate visualization of the posterior cortex.

This additional projection may facilitate intraoperative identification of guidewires positioned close to the posterior cortex, allowing timely repositioning before definitive implant placement. It may be particularly useful in anatomically complex or comminuted fractures where reduction is suboptimal.

The Derriere view is a simple adjunct to standard fluoroscopic imaging that may improve posterior cortical visualization during IT fracture fixation. Further prospective studies are required to determine its reproducibility, clinical effectiveness, and potential to reduce implant malposition and associated complications.

## Introduction

Intertrochanteric (IT) hip fractures are common in elderly patients [1], and cephalomedullary nailing is frequently used for surgical fixation. Fixation failure remains a concern, with superior cut-out and central cut-through representing established modes of failure distinct from posterior femoral neck cortical breach. In the retrospective cohort reported by Raghuraman et al. [2], fixation failure occurred in 5.4% of patients treated with proximal femoral nail antirotation. Suboptimal fracture reduction and implant positioning are recognized contributing factors [2,3].

Standard anteroposterior (AP) and lateral fluoroscopic views may inadequately delineate the posterior femoral neck cortex, particularly when obscured by the targeting jig, greater trochanter, or other instrumentation. This can limit assessment of guidewire position relative to the posterior cortical boundary.

Supplementary fluoroscopic projections for assessing the femoral neck cortex have previously been described, including the 40° sequential rollover view by Aibinder et al. [4], modified projections by Kumar et al. [5], the 150° tangential projection by Zhang and Tang [6], and cadaveric projection analysis by Zhu et al. [7]. These techniques were predominantly described for femoral neck fracture fixation rather than cephalomedullary nailing of IT fractures.

This technical report describes the Derriere view, an approximately 40° cranial oblique projection from the standard lateral position, used during cephalomedullary nailing with the targeting jig in situ. Its purpose is to improve visualization of the posterior femoral neck cortex and supplement standard fluoroscopic assessment of guidewire position.

## Technical report

Material and methodology

The morphology of the proximal femur is complex. Bonneau et al. [8] demonstrated that the femoral neck is not a simple cylindrical structure but is better represented by successive elliptical cross-sections with anteroposterior flattening. Yin et al. [9] described assessment of the femoral neck axis using an axial oblique CT slice passing through the centers of the femoral head and neck. Along this axis, the anterior and posterior cortices are asymmetrical, with the posterior cortex being relatively more concave and the anterior cortex more linear. Nakanishi et al. [10] further demonstrated that the posterior wall lies closer to the femoral neck axis than the anterior wall at the narrowest level of the neck. Consequently, a guidewire that appears centrally positioned on conventional fluoroscopic views may still lie close to the posterior cortical boundary. This anatomical asymmetry, combined with incomplete visualization of the posterior cortex on standard lateral views, provides the rationale for an additional oblique projection that more clearly delineates the posterior cortex and allows assessment of guidewire proximity to it. We therefore propose the “Derriere view” as an adjunctive intraoperative fluoroscopic projection.

**Figure 1:**
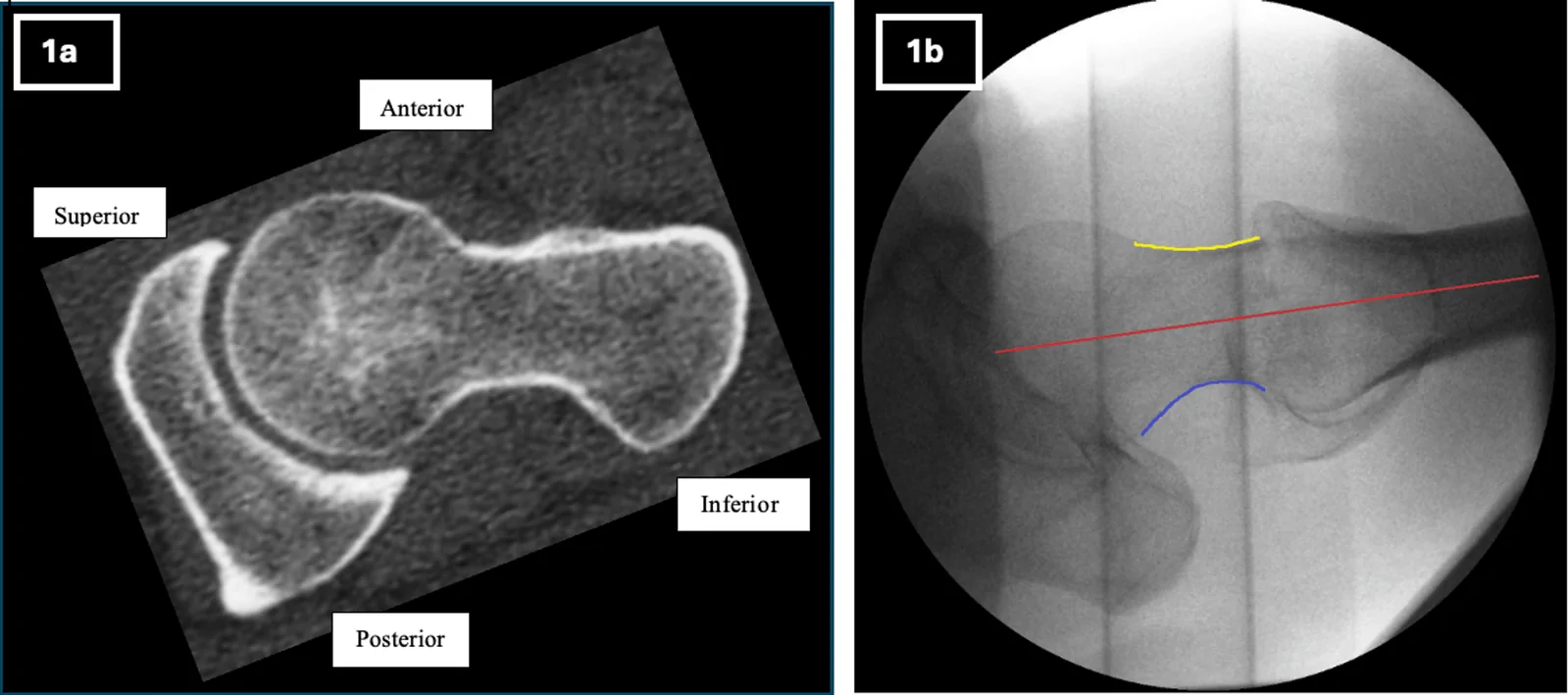
CT and fluoroscopic illustration of the femoral neck anatomy. Figure 1a: CT image of the femoral neck at its narrowest level, with anatomical orientation labeled. Figure 1b: Intraoperative fluoroscopic image with superimposed annotations demonstrating the anterior femoral neck cortex (yellow), posterior femoral neck cortex (blue), and an illustrative guidewire trajectory (red) through the femoral head and neck.

Surgical technique

To ensure implants are inserted precisely at the most optimal position, the authors propose a supplementary intraoperative fluoroscopic view for the posterior femoral neck cortex called the ‘Derriere view’.

To improve intraoperative assessment of guidewire position, several supplementary fluoroscopic projections have previously been described to visualize the posterior femoral neck cortex, predominantly in the context of cannulated screw fixation of femoral neck fractures. These include sequential rollover, modified oblique, and tangential fluoroscopic projections. In contrast, the Derriere view described in this technical report applies an approximately 40° cranial oblique projection from the standard lateral position during cephalomedullary nailing of intertrochanteric fractures, with the targeting jig and instrumentation in situ. The view is intended to provide additional visualization of the posterior femoral neck cortex when this boundary is inadequately delineated on conventional projections. In the representative case presented, the Derriere view demonstrated the guidewire in close proximity to the posterior cortical boundary and prompted intraoperative repositioning. This report describes the technical application of the projection rather than a formal clinical series or validation study. Further anatomical and prospective clinical evaluation is required to determine its reproducibility, diagnostic accuracy, and clinical utility.

The patient was positioned supine on a fracture table, with the injured limb positioned for standard cephalomedullary nailing. Following fracture reduction and nail insertion, standard AP and lateral fluoroscopic views were obtained. The standard lateral projection was defined as the 0° reference position for subsequent C-arm rotation.

A 20° cranial oblique projection from the standard lateral position was first obtained to provide a more direct lateral view of the femoral neck. However, the posterior femoral neck cortex may remain obscured by superimposition of the targeting jig, greater trochanter, nail, and other instrumentation (Figure 2).

**Figure 2:**
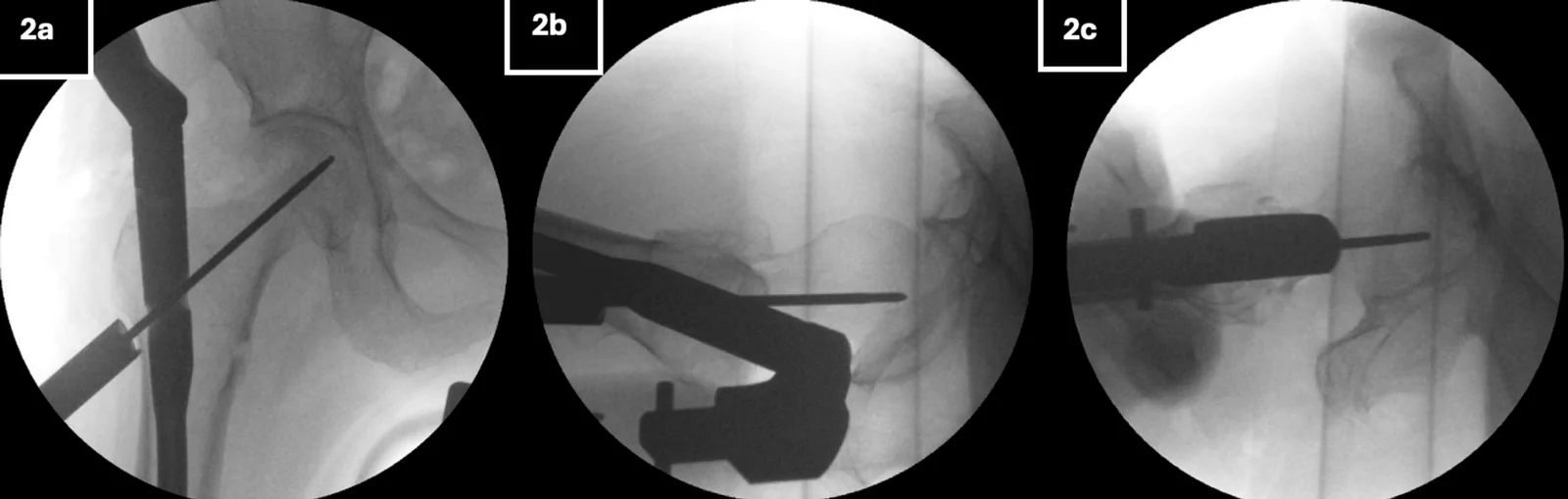
Standard fluoroscopic views used during cephalomedullary nailing. Figure 2a: Anteroposterior (AP) view. Figure 2b: Standard lateral view. Figure 2c: 20° oblique lateral view used to obtain a more direct lateral projection of the femoral neck. In these conventional projections, the posterior femoral neck cortex may remain inadequately visualized because of superimposition of the nailing jig and greater trochanter.

To obtain the Derriere view, the C-arm is returned to the standard lateral position and then rotated approximately 40° cranially from the standard lateral (0°) position (Figure 3a). Thus, the stated 40° refers to the angle relative to the standard lateral view and not an additional 40° from the 20° oblique position. No additional cephalocaudal tilt is applied. A satisfactory Derriere view is obtained when the posterior femoral neck cortex is clearly delineated with reduced superimposition from the targeting jig and proximal femoral anatomy, allowing the relationship between the guidewire and posterior cortical boundary to be assessed (Figure 3b). The 40° angle serves as an approximate starting point rather than a fixed anatomical angle. Minor adjustment may be required according to patient anatomy, femoral anteversion, fracture reduction, and intraoperative positioning until the posterior cortex is adequately visualized. If the guidewire is considered suboptimally positioned relative to the posterior cortex, it can be revised before definitive implant insertion. Written informed consent was obtained from the patient for publication of this case report and the accompanying clinical images. All figures are original images obtained from the authors' own case and have been anonymized to protect patient confidentiality.

**Figure 3:**
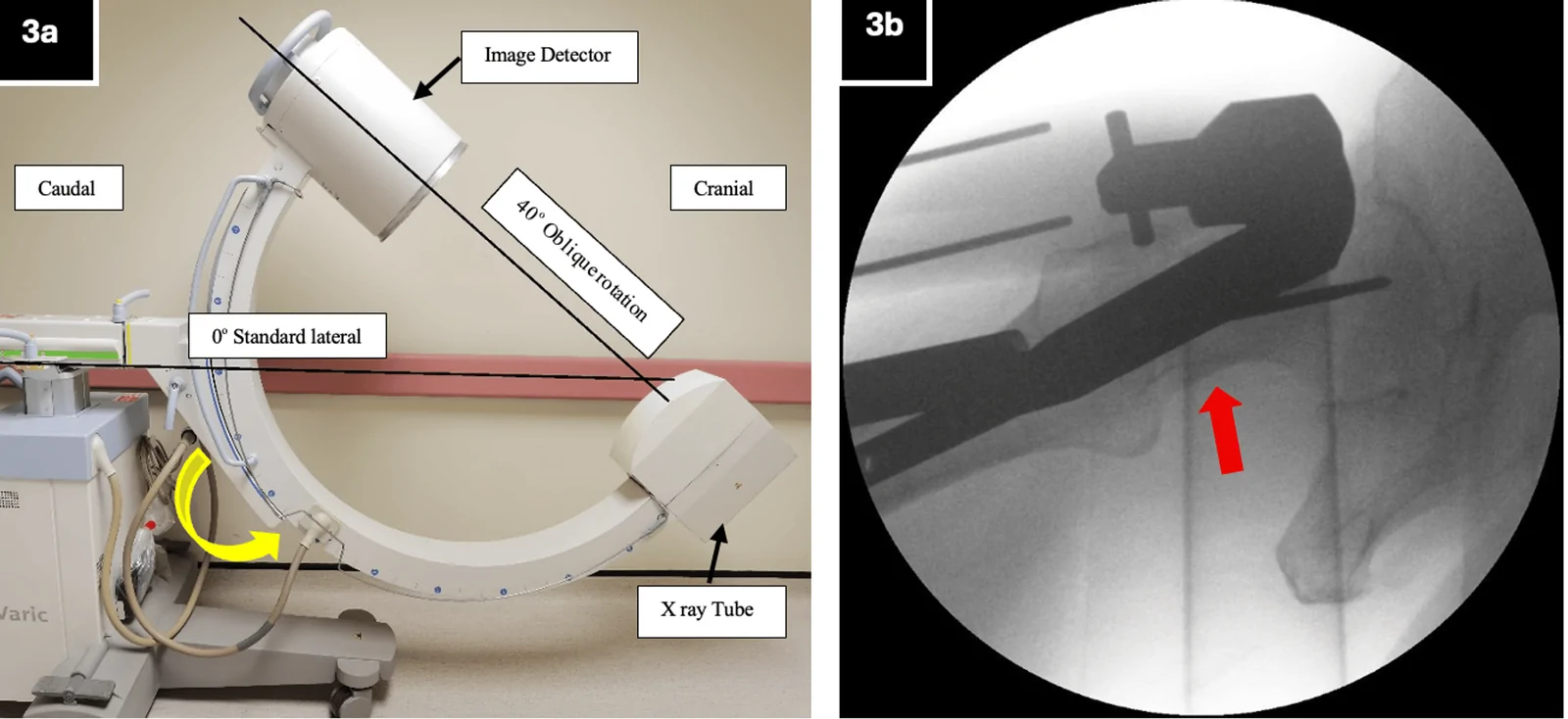
C-arm positioning and fluoroscopic appearance of the Derriere view. Figure 3a: C-arm setup for obtaining the Derriere view. The standard lateral projection is defined as the 0° reference position. From this position, the C-arm is rotated approximately 40° cranially in the direction indicated by the yellow arrow. The patient orientation, standard lateral reference, direction and magnitude of rotation, X-ray tube, and image detector are labeled in the schematic. Figure 3b: Derriere fluoroscopic view demonstrating the posterior femoral neck cortex (red arrow) and its relationship to the guidewire. This projection provides additional visualization of the posterior cortical boundary when it is inadequately delineated by superimposition of the proximal femoral anatomy and instrumentation on conventional views.

## Discussion

During cephalomedullary nailing of the femur, the targeting jig attached to the nail may obscure the guidewire for the proximal blade as it is advanced into the femoral neck. Consequently, conventional fluoroscopic views may provide limited visualization of the guidewire relative to the asymmetrical anterior and posterior femoral neck cortices, making it difficult to confirm a center-center guidewire position relative to the femoral neck axis.

Risk factors for fracture fixation failure: Kaufer [11] stated that stability after fracture fixation is determined by five principal factors: bone quality, fracture comminution, fracture reduction quality, implant selection and implant placement. Of these, implant selection, fracture reduction, and implant placement are potentially modifiable surgical factors. Chang [12] in 2020 covered the topic of “Intertrochanteric Femur Fracture Treatment in Asia” extensively. It concluded that the quality of the fracture reduction and implant positioning, including tip-apex distance, has a direct impact on the stability of the fixation postoperatively. 

Additional C-arm projections are commonly used intraoperatively to improve fluoroscopic assessment. Chen SY [13] proposed an additional view to evaluate the anteromedial cortex during reduction by using an anteromedial oblique view of 30° to eliminate the occlusive overlapping shadows.

Moreover, not all IT fractures could be reduced anatomically. In cases where anatomical reduction was not possible, a positive or neutral lateral cortical position on intra-operative fluoroscopy is a reliable predictor of final cortical support and may help prevent axial sliding of the proximal fragment [14]. In these cases, the lateral view or true lateral view will overestimate the distance between the center of the femoral head and the true position of the posterior cortex and increase the risk of implant blowout of the posterior cortex. Hoffmann et al. [15], in a cadaveric study of cannulated screw fixation for femoral neck fractures, demonstrated that posterior-cranial screw breach with core extrusion could occur despite standard AP and lateral fluoroscopic views suggesting that the screws remained contained within the femoral neck cortices. Although their study involved cannulated screw fixation of femoral neck fractures rather than cephalomedullary fixation of intertrochanteric fractures, the finding illustrates a relevant fluoroscopic limitation: standard orthogonal views may not reliably demonstrate implant proximity to or breach of the posterior femoral neck cortex. This principle provides indirect support for the use of an additional fluoroscopic projection when posterior cortical visualization is inadequate during cephalomedullary nailing.

An example of a comminuted displaced reverse oblique IT fracture is shown in Figure 4a. Despite attempted reduction, significant comminution made anatomical alignment of both the anterior and posterior cortices difficult to achieve. In the illustrative case, due to superimposition of the nailing jig, it was difficult to confirm central guidewire positioning using the standard lateral (Figure 4b) and 20° oblique lateral views (Figure 4c) alone. The Derriere view provided clearer visualization of the posterior cortex and demonstrated that the projected guidewire was positioned close to the posterior cortical boundary (Figure 5a). The guidewire was subsequently revised, and its improved position was confirmed intraoperatively using the standard AP and lateral fluoroscopic views together with the Derriere view, which demonstrated satisfactory positioning away from the posterior cortex (Figure 5b). Enlarged annotated images are provided to demonstrate the guidewire and posterior femoral neck cortex before and after adjustment.

**Figure 4:**
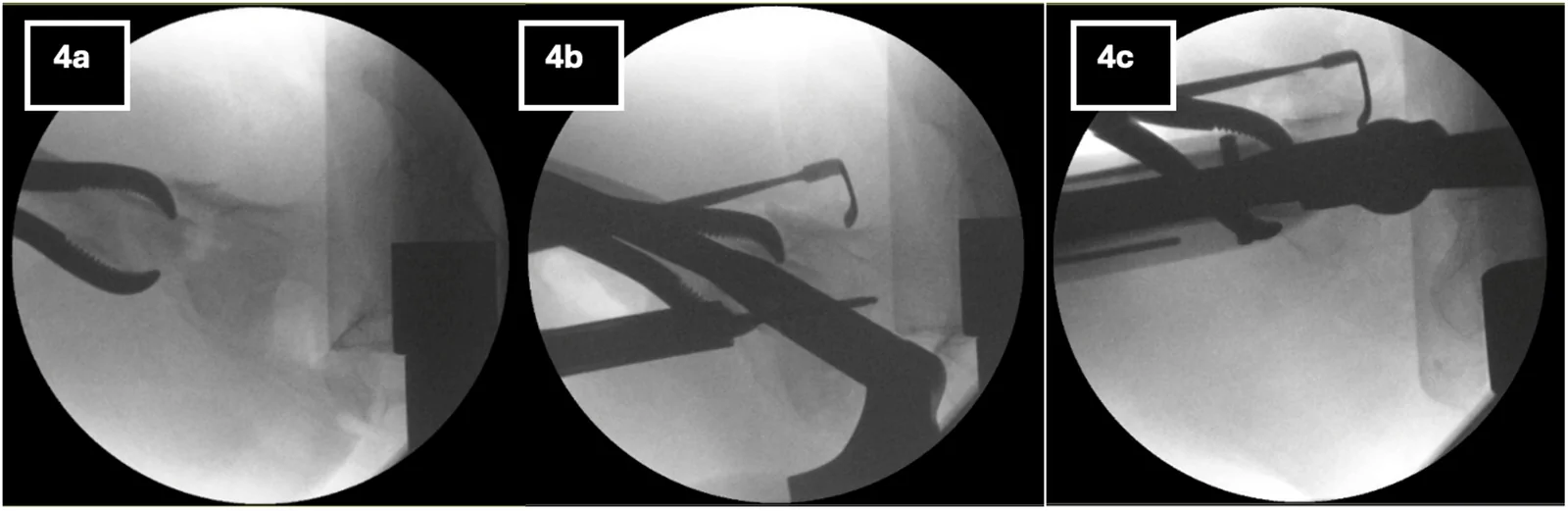
Intraoperative fluoroscopic views of a comminuted reverse oblique intertrochanteric femur fracture. Figure 4a: Anteroposterior (AP) fluoroscopic view demonstrating a comminuted, displaced, reverse oblique intertrochanteric fracture. Figure 4b: Standard lateral fluoroscopic view of the hip. Figure 4c: 20° oblique lateral fluoroscopic view, used to obtain a more direct lateral projection of the femoral neck. The posterior femoral neck cortex remains inadequately visualized because of superimposition of the nailing jig, intramedullary nail, and instruments used to maintain fracture reduction.

**Figure 5:**
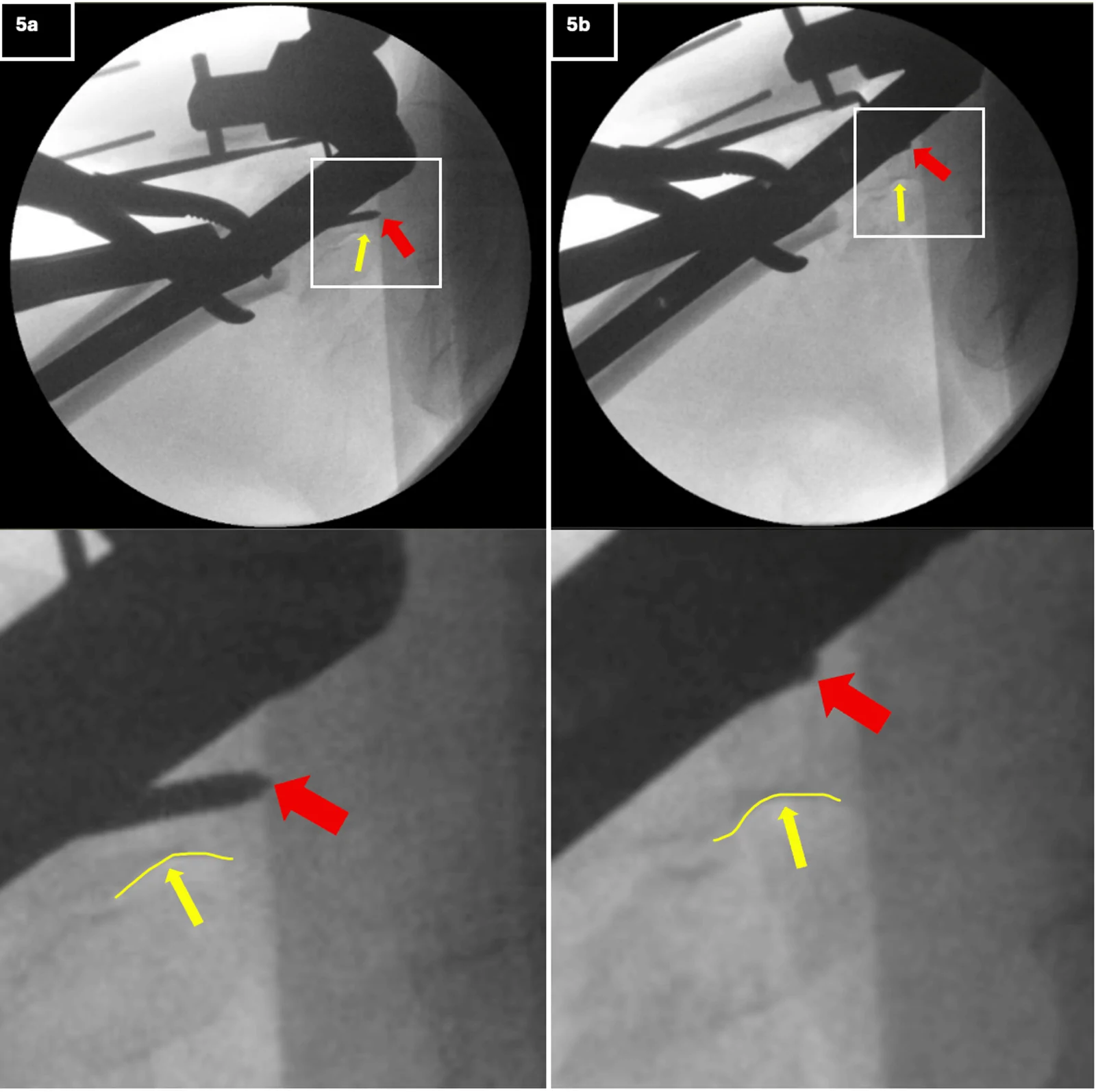
Derriere views demonstrating guidewire position relative to the posterior femoral neck cortex. Figure 5a: Derriere view before repositioning, with the initial guidewire (red arrow) and posterior femoral neck cortex (yellow arrow) separately annotated. An enlarged inset is provided to demonstrate their relationship more clearly. Figure 5b: Derriere view following guidewire repositioning, with the revised guidewire position (red arrow) and posterior femoral neck cortex (yellow arrow) separately annotated. An enlarged inset is provided to facilitate assessment despite the superimposition of the surrounding instrumentation.

Although a similar projection may be recreated by manually lifting the jig during acquisition of a lateral view, this maneuver is not recommended because manipulation of the construct at this stage may risk loss of fracture reduction.

This technical report has several limitations. It describes a single illustrative case and does not provide comparative or quantitative data demonstrating superiority over conventional fluoroscopic views. The additional projection may also increase radiation exposure and operative time and requires familiarity with C-arm positioning, introducing a potential learning curve. Furthermore, the optimal projection angle may vary according to patient anatomy, femoral anteversion, fracture reduction, and positioning. Prospective studies are required to assess reproducibility, additional radiation and operative time, guidewire positioning, and clinical outcomes.

## Conclusions

The Derriere view is a potentially useful adjunctive intraoperative fluoroscopic projection for assessing the posterior femoral neck cortex during cephalomedullary nailing of intertrochanteric fractures. In the illustrative case presented, the projection provided additional visualization of the posterior cortical boundary and prompted guidewire repositioning. However, its reproducibility, diagnostic accuracy, superiority over conventional fluoroscopic views, and effect on implant-related complications or clinical outcomes have not been established. Further anatomical and prospective clinical studies are required to evaluate the technique.
